# Association between genetic polymorphisms and gestational diabetes mellitus susceptibility in a Chinese population

**DOI:** 10.3389/fendo.2024.1397423

**Published:** 2024-11-26

**Authors:** Qiaoli Zeng, Jia Liu, Xin Liu, Na Liu, Weibiao Wu, Ray Gyan Watson, Dehua Zou, Yue Wei, Runmin Guo

**Affiliations:** ^1^ Department of Internal Medicine, Shunde Women and Children’s Hospital (Maternity and Child Healthcare Hospital of Shunde Foshan), Guangdong Medical University, Foshan, Guangdong, China; ^2^ Key Laboratory of Research in Maternal and Child Medicine and Birth Defects, Guangdong Medical University, Foshan, Guangdong, China; ^3^ Matenal and Child Research Institute, Shunde Women and Children’s Hospital (Maternity and Child Healthcare Hospital of Shunde Foshan), Guangdong Medical University, Foshan, Guangdong, China; ^4^ Department of Pediatrics, Shunde Women and Children’s Hospital (Maternity and Child Healthcare Hospital of Shunde Foshan), Guangdong Medical University, Foshan, Guangdong, China; ^5^ Medical Genetics Laboratory, Shunde Women and Children’s Hospital (Maternity and Child Healthcare Hospital of Shunde Foshan), Guangdong Medical University, Foshan, Guangdong, China; ^6^ School of Pharmacy, Macau University of Science and Technology, Macao, Macao SAR, China; ^7^ Guangdong Engineering Research Center of Chinese Medicine and Disease Susceptibility, Jinan University, Guangzhou, Guangdong, China; ^8^ Department of Ultrasound, Shunde Women and Children’s Hospital (Maternity and Child Healthcare Hospital of Shunde Foshan), Guangdong Medical University, Foshan, Guangdong, China

**Keywords:** gestational diabetes mellitus, rs1111875, rs5015480, rs11705701, rs4402960, rs9939609, case-control study

## Abstract

**Background:**

Although the association between HHEX, IGF2BP2, and FTO polymorphisms and the risk of GDM has been investigated in several studies, the findings have been inconsistent across different populations. The study aimed to investigate the association between genetic polymorphisms and GDM risk in a Chinese population.

**Methods:**

502 control volunteers and 500 GDM patients were enrolled. IGF2BP2 rs11705701 and rs4402960, FTO rs9939609, and HHEX rs1111875 and rs5015480 were all genotyped using the SNPscan™ genotyping assay. The independent sample t-test, logistic regression, and chi-square test were used to assess the variations in genotype and allele and their relationships with the risk of GDM. The blood glucose level, gestational week of delivery, and newborn weight were compared using a one-way ANOVA.

**Results:**

After adjusting for confounding factors, the results show that the rs1111875 heterozygous (OR=1.370; 95% CI: 1.040-1.805; *P* = 0.025) and overdominant (OR=1.373; 95% CI: 1.049-1.796; *P* = 0. 021) models are significantly associated with an increased risk of GDM, especially for the age ≥ 30 years group: heterozygote (OR=1.646; 95% CI: 1.118-2.423; *P*=0.012) and overdominant (OR=1.553; 95% CI: 1.064-2.266; *P* = 0.022) models. In the age ≥ 30 years, the rs5015480 overdominant model (OR=1.595; 95% CI: 1.034-2.459; *P* = 0.035) and the rs9939609 heterozygote model (OR=1.609; 95% CI: 1.016-2.550; *P*=0.043), allele (OR=1. 504; 95% CI: 1.006-2.248; P = 0.047), dominant model (OR=1.604; 95% CI: 1.026-2.505; *P* = 0.038), and overdominant model (OR=1.593; 95% CI: 1.007-2.520; *P* = 0.047) were associated with a significantly increased risk of GDM; Additionally, people with the TC genotype of rs1111875 had a substantially higher 1-hour blood glucose level than TT genotype (*P* < 0.05). The results of the meta-analysis showed that the A allele of rs11705701 was associated with an increased risk of diabetes mellitus (*P* < 0.05).

**Conclusion:**

The study indicates that the TC genotype of rs1111875 is linked to a higher risk of GDM, particularly in women aged 30 years or older. Additionally, rs5015480 and rs9939609 were significantly associated with GDM in the same age group. These SNPs may therefore be more closely linked to GDM in older mothers.

## 1 Introduction

Gestational diabetes mellitus (GDM) as diabetes diagnosed during pregnancy that is not clearly overt diabetes. It is a common disease in pregnancy that is determined by the first diagnosis of hyperglycemia (1, 2). It is associated with hypertensive disorders of pregnancy, macrosomia, cesarean section, and neonatal complications (3). The prevalence of GDM ranges from 1.8% to 25.1% globally (4). GDM is a multifactorial complex metabolic disorder influenced by genetic and environmental factors, similar to type 2 diabetes mellitus (T2DM). Genetics is crucial to GDM and is not easily modifiable through intervention (5). Studies have demonstrated that several genes linked to T2DM risk are also related to GDM, and polymorphisms in HHEX, IGF2BP2, and FTO have been linked to decreased β-cell function and diabetes risk (6–11).

The hematopoietically expressed homeobox (HHEX) gene is situated in the 270 kb linkage disequilibrium (LD) region of human chromosome 10, q23.33. It plays a regulatory role in insulin secretion and diabetes mellitus. The LD block comprises three genes: the kinase family member 11 gene, the insulin- degrading enzyme gene, and HHEX. The regions rs1111875 and rs5015480 are closest to HHEX, which has been linked to diabetes mellitus (DM), and are situated close to the LD region (12–15). Moreover, previous studies have shown that polymorphisms in the insulin-like growth factor 2 mRNA binding protein 2 (IGF2BP2) gene may be a risk factor for obesity and T2DM (10, 16). IGF2BP2 belongs to a family of messenger ribonucleic acid-binding proteins that regulate the translation of IGF2 (17). IGF2BP2 promotes the release of insulin and is essential for the growth and development of pancreatic β-cells. Alpha-ketoglutarate-dependent dioxygenase, or FTO, is involved in energy balance, and lipid and carbohydrate metabolism. T2DM and obesity have been associated with variations in the FTO gene (11, 18–20).

Some research works have investigated the relationship between the risk of GDM and HHEX rs1111875 and rs5015480, IGF2BP2 rs11705701 and rs4402960, and FTO rs9939609 (21, 22). Nevertheless, the outcomes have displayed variability. The objective of this study was to examine the relationship between gene polymorphisms (rs1111875 and rs5015480, rs11705701 and rs4402960, and rs9939609) and the risk of GDM in the Chinese population. Additionally, our objective was to examine the connections between gene polymorphisms and clinical parameters, such as glycemia, week of gestation, and newborn weight.

## 2 Materials and methods

### 2.1 Study subjects

With a total of 1002 participants, the study involved 500 patients with GDM and 502 healthy pregnant women as controls. The study protocol for this research was approved by the Ethics Committee of Shunde Women and Children’s Hospital at Guangdong Medical University (the ethical approval number: 2020072), and subjects were selected based on specific criteria: (i) Han ethnicity; (ii) age ≥ 18 years; (iii) voluntary informed consent; (iv) never diagnosed with diabetes; (v) no glucose-lowering medication; and (vi) no pregnancy complications. Participants were excluded if they had previously been diagnosed with diabetes, were under 18 years of age, had pregnancy complications, or were taking glucose-lowering medication. A total of 1002 pregnant Chinese Han women provided voluntary informed consent. According to the diagnostic criteria established by the International Association of Diabetes and Pregnancy Study Groups (IADPSG), pregnant women underwent an oral glucose tolerance test (OGTT) between weeks 24 and 28 of gestation. In cases where at least one glucose level measurement equals or exceeds the threshold value, the subject was considered positive for GDM. Subjects with GDM were identified through the assessment of their blood glucose levels: fasting blood glucose (FBG) ≥ 5.1 mmol/L, or 1-hour postprandial glucose (1h-PG) ≥ 10.0 mmol/L, or 2-hour postprandial glucose (2h-PG) ≥ 8.5 mmol/L. Healthy controls were defined as individuals with normoglycemic levels. The study was carried out in compliance with the principles of the Declaration of Helsinki.

### 2.2 Data collection

In the study, general clinical information was compiled, encompassing age, ethnicity, height, systolic blood pressure (SBP), diastolic blood pressure (DBP), prepregnancy weight, and parity (primipara or multipara). The prepregnancy body mass index (pre-BMI, Kg/m^2^) was computed as the ratio of prepregnancy weight (Kg) to the square of the height (m^2^). The pre-BMI categorizes Chinese individuals into the following groups based on their weight: Obesity is defined as having a BMI equal to or greater than 28 Kg/m^2^, overweight falls within the range of 24 Kg/m^2^ to less than 28 Kg/m^2^, normal weight is classified between 18.5 Kg/m^2^ and less than 24 Kg/m^2^, and underweight is indicated by a BMI of less than 18.5 Kg/m^2^.

### 2.3 SNP genotyping

Extraction of genomic DNA was conducted utilizing the QIAamp DNA blood kit (Qiagen, Germany), followed by genotyping of individual SNPs through the SNPscan method. The resulting raw data were acquired using an ABI3730XL sequencer and processed using GeneMapper 4.1 software (Applied Biosystems, USA) by Genesky Technologies Inc. (Shanghai, China). Rigorous quality control protocols were enforced to ensure the precision of the genotyping results.

### 2.4 Statistical analyses

Statistical analyses were conducted using SPSS 20.0 software (SPSS, Chicago, IL, USA). Continuous variables were compared using an independent sample t-test, presenting the results as mean ± standard deviation. Discontinuous variables, such as Hardy-Weinberg equilibrium (HWE) in the control group, were analyzed using chi-square tests. The association between SNP and GDM risk was evaluated through binary logistic regression analysis, adjusting for potential confounders like pre-BMI, age, parity, and blood pressure. The results were expressed as a odds ratio (OR) with a 95% confidence interval (CI). We used one-way ANOVA to analyze the correlation between SNP and blood glucose levels, gestational week of labor, and neonatal weight. For significant one-way ANOVA results, we continued with the least significant difference (LSD) comparisons. Subgroup analyses for age and pre-BMI were also performed. Heterogeneity was estimated using Q-test and I^2^ test. No heterogeneity was defined as I^2^ < 50% and *P* > 0.1, STATA v.16.0 software (Stata Corporation, Texas, United States) was used to perform heterogeneity analyses. A statistically significant result was observed for bilateral *P* < 0.05.

### 2.5 Meta-analysis

A systematic search was conducted across PubMed, Chinese National Knowledge Infrastructure, and Google Scholar databases using various combinations of the terms rs11705701, Gestational diabetes mellitus (GDM), type 2 diabetes mellitus (T2DM), type 1 diabetes mellitus (T1DM), and prediabetes mellitus (Pre-DM, without any restrictions. Inclusion criteria encompassed case-control or cohort studies investigating the relationship between rs11705701 and GDM, T2DM, T1DM, or Pre-DM, and providing adequate raw data. Studies not meeting diagnostic criteria or exhibiting data deviating from HWE were excluded. Data extraction was overseen by two authors. The meta-analysis, utilizing fixed or random effects models based on heterogeneity levels, was conducted across six genetic models. Publication bias was evaluated through Egger’s and Begg’s tests. All statistical analyses were performed using STATA v.16.0 software (Stata Corporation, TX, USA).

### 2.6 Prediction of transcription factors

The online tool PROMO (https://alggen.lsi.upc.es/home.html) was utilized to investigate whether rs11705701 located in the promoter region of the IGF2BP2 gene impacts the binding sites of transcription factors (23, 24).

## 3 Results

### 3.1 General clinical characteristics of the subjects

In this case-control research, 502 healthy controls and 500 GDM patients were examined. The genotypes of FTO rs9939609, IGF2BP2 rs11705701 and rs4402960, and HHEX rs1111875 and rs5015480 were studied. The clinical baseline data is shown in **Table 1**. In comparison to the control group, the GDM group exhibited substantially higher mean age, pre-BMI, SBP, DBP, and blood glucose levels (*P* < 0.05). Furthermore, there was a significant difference (*P* < 0.05) in the parity between the GDM group and the control group.

**Table 1:** Basic and stratified characteristic of participants of the study.

Variables	Cases (%)	Controls (%)	t/χ2	*P*
Age, year (mean ± SD), overall	31 ± 4	29 ± 4	-8.56	**< 0.001**
<30	27 ± 2	26 ± 3	-3.64	**< 0.001**
≥30	34 ± 3	33 ± 2	-3.14	**0.002**
pre-BMI, Kg/m2, overall	21.51 ± 3.10	20.53 ± 2.58	-5.42	**< 0.001**
<18.5	17.45 ± 0.84	17.60 ± 1.50	0.75	0.453
18.5 ≤ BMI < 24	20.96 ± 1.49	20.67 ± 1.41	-2.63	**0.009**
≥24	26.16 ± 2.84	25.83 ± 3.31	-0.60	0.548
SBP, mmHg, overall	117 ± 11	114 ± 10	-3.53	**< 0.001**
DBP, mmHg, overall	70 ± 8	68 ± 7	-3.23	**0.001**
FBP, mmol/L, overall	4.82 ± 0.64	4.50 ± 0.31	-9.75	**< 0.001**
1h-PG, mmol/L, overall	10.17 ± 1.60	7.66 ± 1.27	-26.22	**< 0.001**
2h-PG, mmol/L, overall	8.91 ± 1.60	6.69 ± 0.99	-25.85	**< 0.001**
Gestational week of labor, overall	38.8 ± 1.12	39.18 ± 2.38	3.20	**0.001**
Neonatal weight, g, overall	3222.94 ± 392.598	3164.01 ± 362.971	-2.47	**0.014**
Parity (n), overall			8.88	**0.003**
Primipara	210 (42)	258 (51.4)		
Multipara	290 (58)	244 (48.6)		

pre-BMI, pre-gestational body mass index; SBP, systolic blood pressure; DBP, diastolic blood pressure; FBP, fasting blood glucose level; 1h-PG, 1 hour blood glucose level; 2h-PG, 2 hour blood glucose level.

### 3.2 The association of SNPs with GDM risk

#### 3.2.1 Overall analysis results

**Figure 1:**
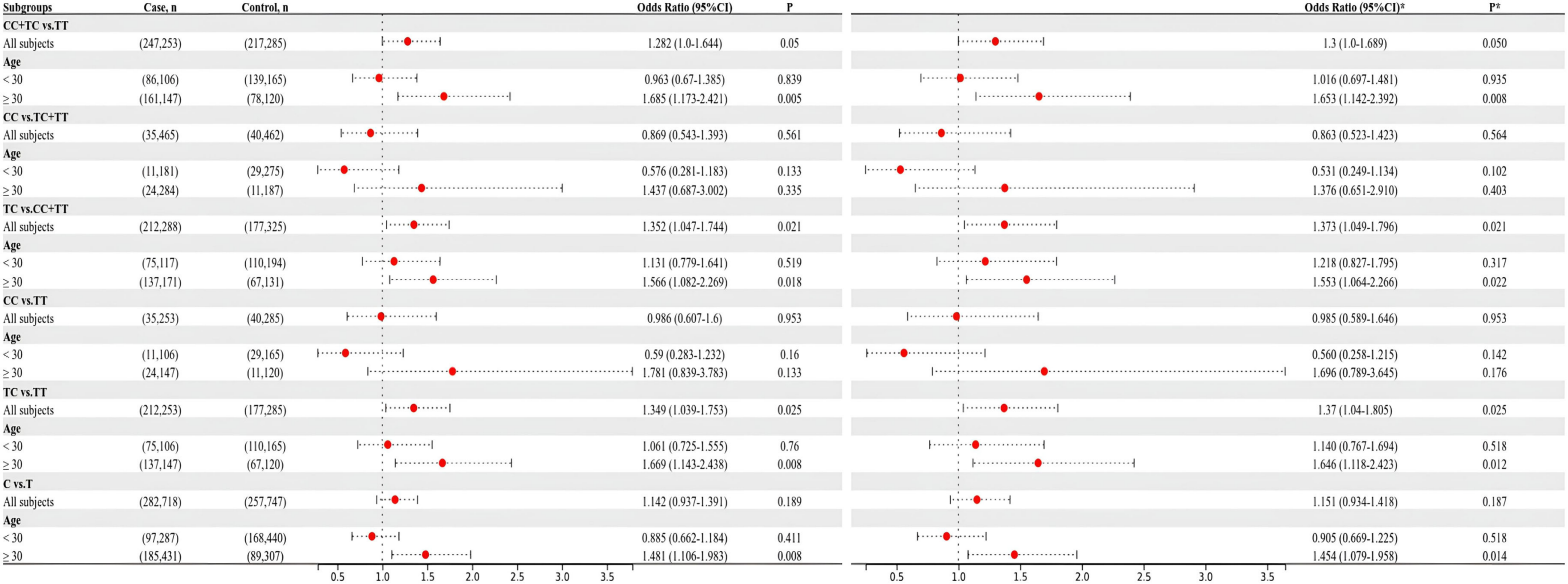
The associations between rs1111875 and GDM risk in different groups. *adjusted.

**Figure 2:**
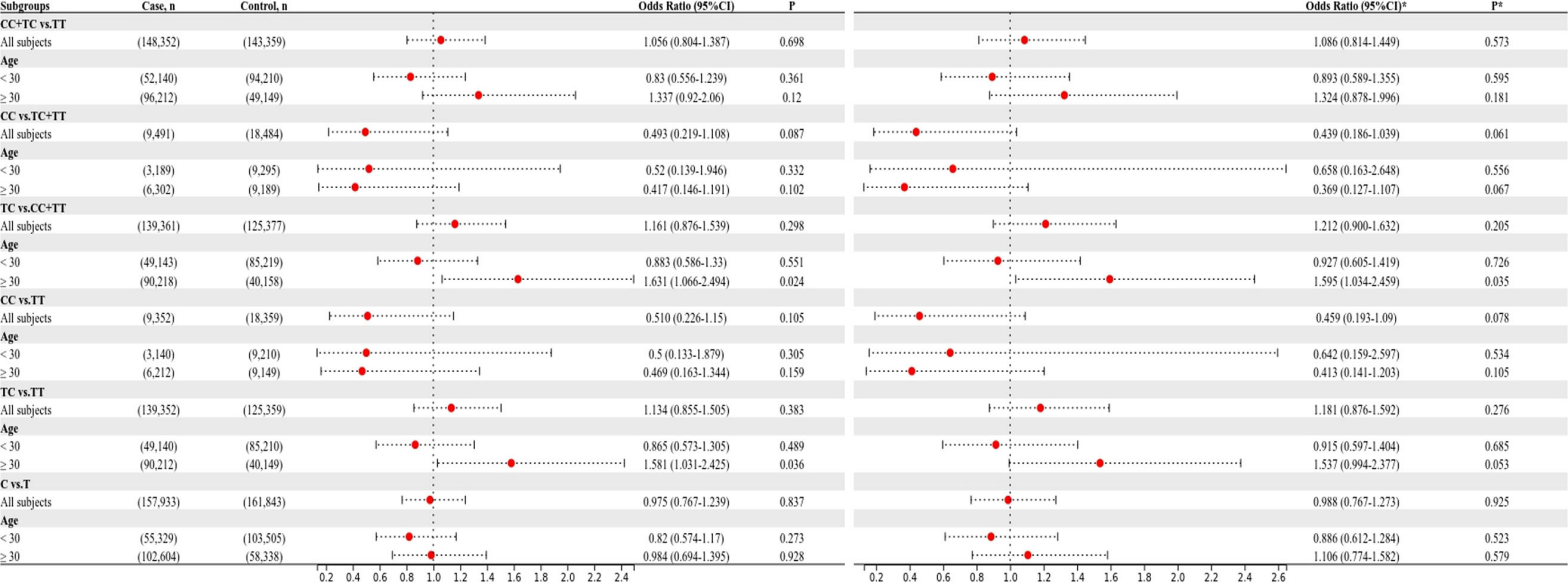
The associations between rs5015480 and GDM risk in different groups. *adjusted.

**Figure 3:**
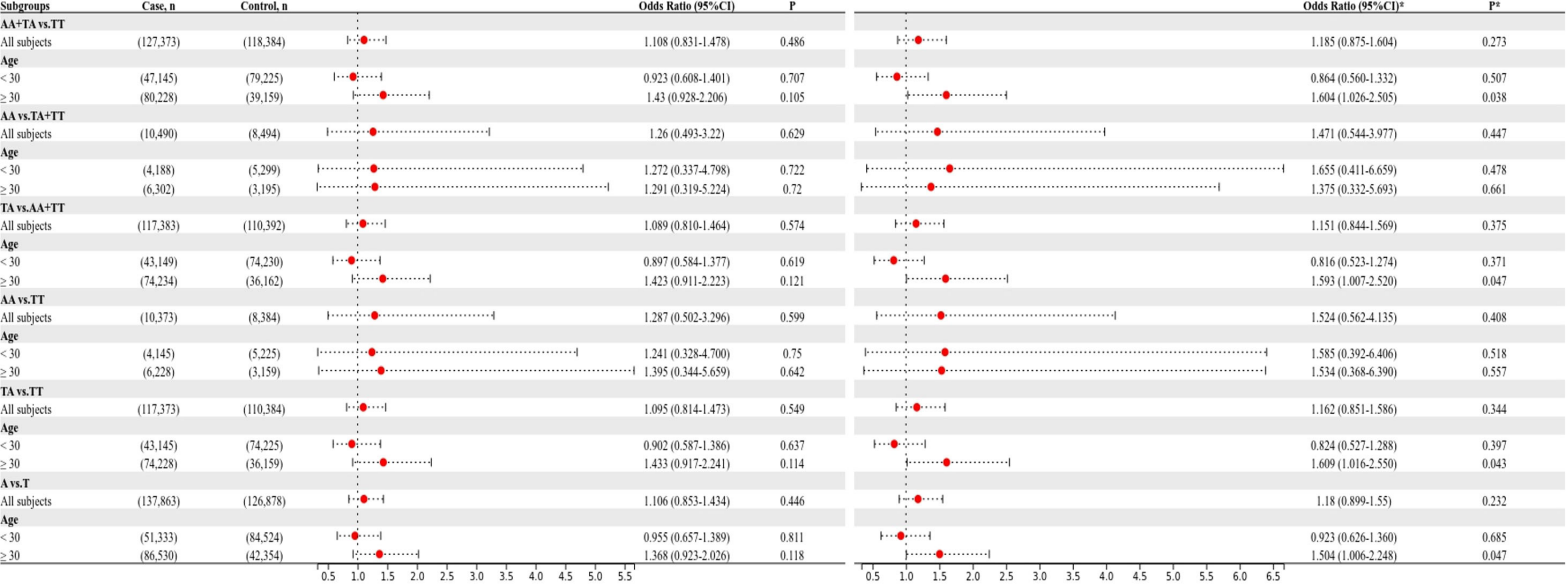
The associations between rs9939609 and GDM risk in different groups. *adjusted.

The HWE analysis and minor allele frequencies (MAF) for the five SNPs in the control group are shown in **Table 2**. Except rs11705701, the results were consistent with HWE (*P* > 0.05), so no comparative analyses were carried out for this SNP. Using six models (homozygous, heterozygous, allele, dominant, recessive, and overdominant) the correlation between genotype and GDM was assessed, and the (unadjusted and adjusted) OR and 95% CI were computed for each SNP. Before adjustment, the results indicated that the rs1111875 heterozygous (TC vs. TT: OR=1.349; 95% CI: 1.039-1.753; *P* = 0.025) and overdominant (TC vs. TT+CC: OR=1.352; 95% CI: 1.047-1.744; *P* = 0.021) models were associated with an increased risk of GDM. After adjusting for pre-BMI, age, DBP, SBP, and parity, the results showed that the rs1111875 heterozygous (TC vs. TT: OR=1.370; 95% CI: 1.040-1.805; *P* = 0.025) and overdominant (TC vs. TT+CC: OR=1.373; 95% CI: 1.049-1.796; *P* = 0.021) models were significantly associated with an increased risk of GDM (**Figure 1**). Nevertheless, rs5015480, rs4402960, and rs9939609 did not significantly correlate with the GDM risk (*P* > 0.05, **Figures 2**, **3**; **Supplementary Table S1**).

**Table 2:** SNPs information and HWE test in the controls.

SNP	GeneName	Min/Maj	Chr. position	Region	Function	MAF	HWE (*P*)
rs1111875	HHEX	C/T	chr10:94462882	3’-flanking	/	0.256	0.101
rs5015480	HHEX	C/T	chr10:94465559	3’-flanking	/	0.16	0.097
rs4402960	IGF2BP2	T/G	chr3:185511687	intron2	/	0.246	0.15
rs11705701	IGF2BP2	A/G	chr3:185544309	5’-flanking	/	0.108	< 0.001
rs9939609	FTO	A/T	chr16:53820527	intron1	/	0.126	1.00

HWE, Hardy–Weinberg equilibrium; Min, minor allele; Maj, major allele; MAF, frequency of minor allele.

#### 3.2.2 Stratified analysis results

The relationship between the four SNPs and the risk of GDM was next examined using stratified analysis based on age or pre-BMI. In the group with an age of 30 or above, before adjustment, the GDM risk was significantly higher in the rs1111875 dominant (CC+TC vs. TT: OR=1.685; 95% CI: 01.173-2.421; *P* = 0.005), overdominant (TC vs. TT+CC: OR=1.566; 95% CI: 1.082-2.269; *P* = 0.018), heterozygote (TC vs. TT: OR=1.669; 95% CI: 1.143-2.438; *P* = 0.008) and allele (C vs. T: OR=1.481; 95% CI: 1.106-1.983; *P* = 0.008) models (**Figure 1**); A significantly higher risk of GDM was seen in the rs5015480 heterozygote (TC vs. TT: OR=1.581; 95% CI: 1.031-2.425; *P* = 0.036) and overdominant (TC vs. TT+CC: OR=1.631; 95% CI: 1.066-2.494; *P* = 0.024) models (**Figure 2**); There was no discernible association between rs9939609, rs4402960 and the risk of GDM (*P* > 0.05, **Figure 3**; **Supplementary Table S1**). After adjusting for pre-BMI, age, SBP, the results of the rs1111875 heterozygote (TC vs. TT: OR=1.646; 95%CI:1.118-2.423; *P* = 0.012) and overdominant (TC vs. TT+CC: OR=1.553; 95% CI: 1.064-2.266; *P* = 0.022), allele (C vs. T: OR=1.454; 95% CI: 1.079-1.958; *P* = 0.014) and dominant (CC+TC vs. TT: OR=1.653; 95% CI: 1.142-2.392; *P* = 0.008) models remained significantly associated with increased GDM risk (**Figure 1**); The rs5015480 overdominant (TC vs. TT+CC: OR=1.595; 95% CI: 1.034-2.459; *P* = 0.035) model showed a significantly increased GDM risk (**Figure 2**). The rs9939609 heterozygote (TA vs. TT: OR=1.609; 95% CI: 1.016-2.550; *P* = 0.043), allele (A vs. T: OR=1.504; 95% CI: 1.006-2.248; *P* = 0.047), dominant (AA+TA vs. TT: OR=1.604; 95% CI: 1.026-2.505; P = 0.038) and overdominant (TA vs. TT+AA: OR=1.593; 95% CI: 1.007-2.520; *P* = 0.047) models showed a significantly increased GDM risk (**Figure 3**); Nevertheless, rs4402960 did not significantly correlate with the risk of GDM (*P* > 0.05, **Supplementary Table S1**). In additon, In subjects less than 30 years of age, pre-BMI < 18.5, 18.5 ≤ pre-BMI <24, and pre-BMI ≥ 24 groups, no significant correlation with GDM risk was found for any SNP (*P* > 0.05, **Figures 1**-**3**; **Supplementary Table S1**; **Supplementary Figures S1–S3**).

### 3.3 Heterogeneity analysis

**Figure 4:**
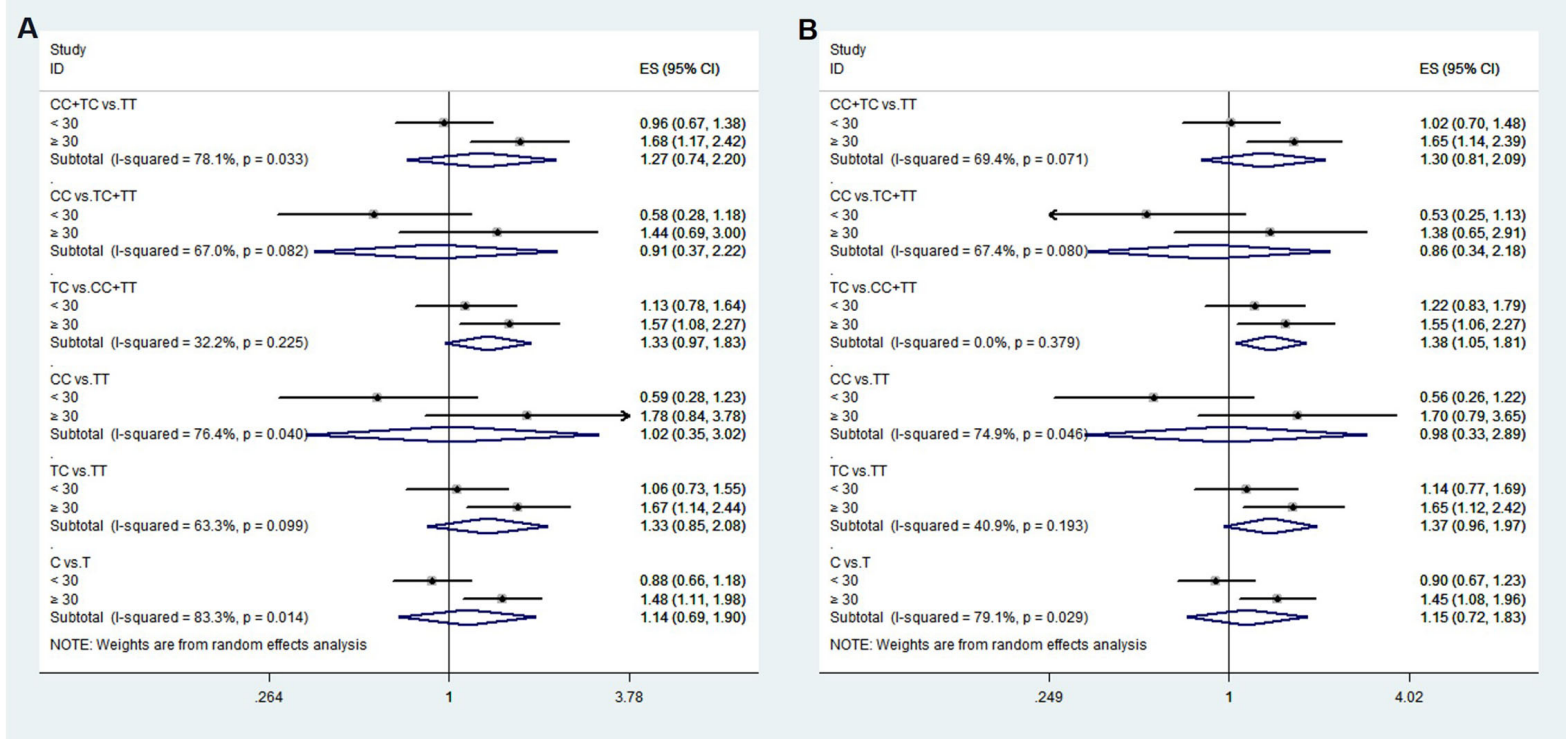
Heterogeneity analysis of various genetic models of rs1111875 among different age groups. **(A)** Unadjusted results, **(B)** Adjusted results.

**Figure 5:**
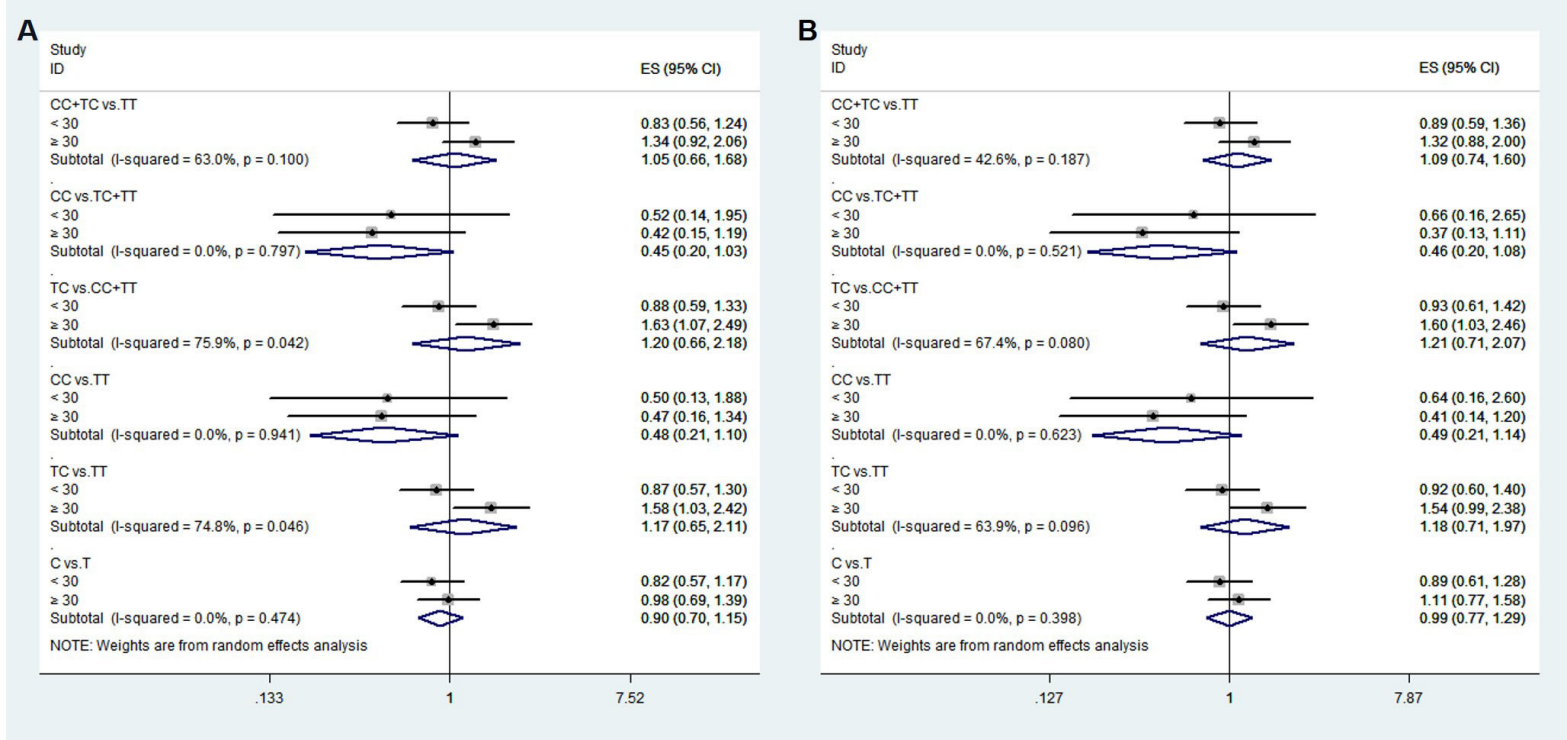
Heterogeneity analysis of various genetic models of rs5015480 among different age groups. **(A)** Unadjusted results, **(B)** Adjusted results.

**Figure 6:**
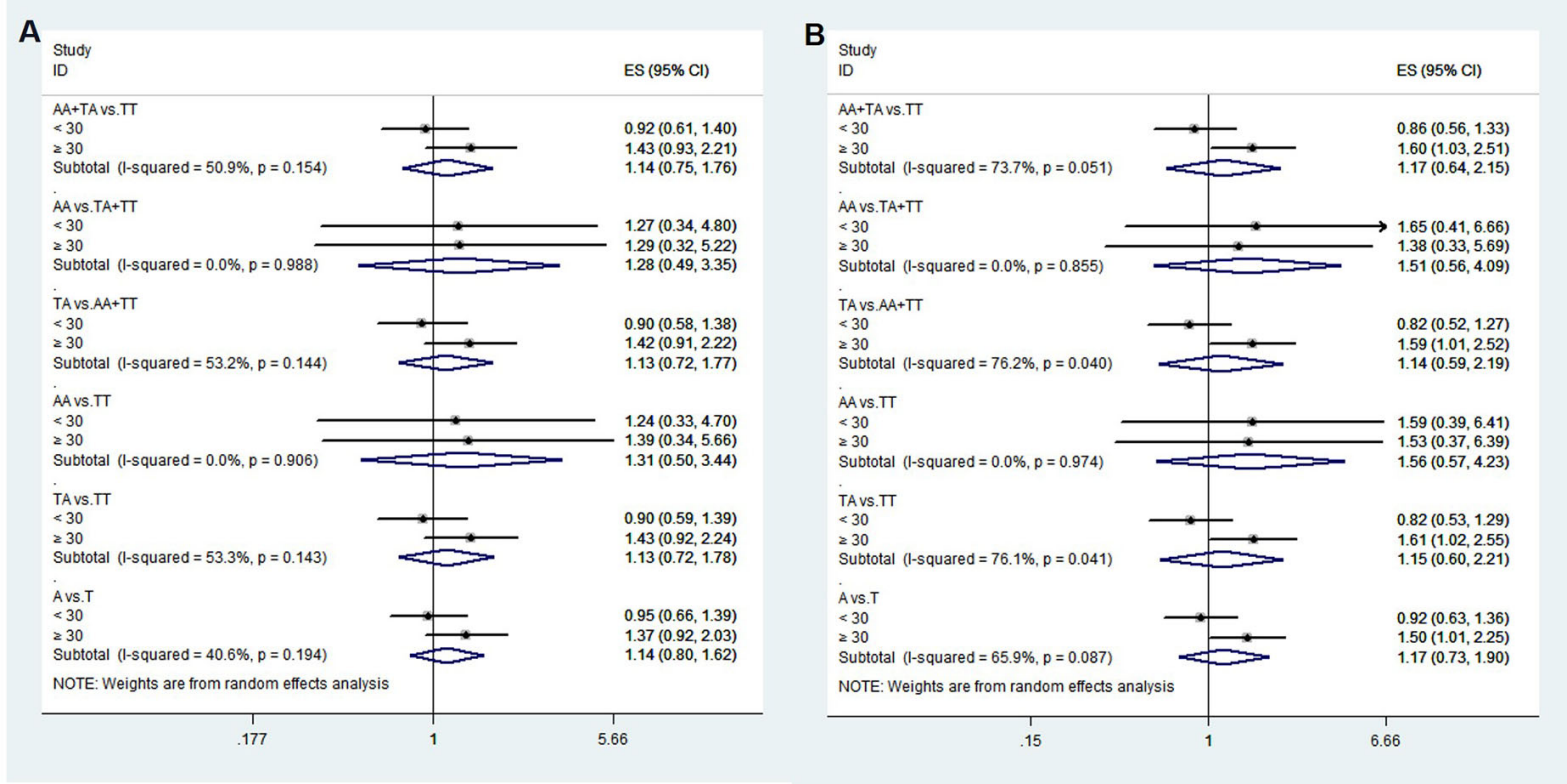
Heterogeneity analysis of various genetic models of rs9939609 among different age groups. **(A)** Unadjusted results, **(B)** Adjusted results.

To confirm that the aforementioned associations were related to age rather than differences between different age groups, we conducted a heterogeneity analysis across different age groups. We found that after adjusting for confounding factors, the heterogeneity among different age groups in various genetic models for rs1111875 and rs5015480 was reduced compared to before adjustment (**Figures 4**, **5**), especially for the overdominant (TC Vs. CC+TT: I^2^ = 0.0%, *P* = 0.379) and heterozygous (TC Vs. TT: I^2^ = 40.9%, *P* = 0.193) models of rs1111875 (**Figure 4**), where no significant heterogeneity was observed. This strongly suggests that the association of rs1111875 with an increased risk of GDM is related to age, particularly in pregnant women aged 30 or older. The high heterogeneity observed across different age groups for other SNPs genetic models may be related to insufficient sample size or other confounding factors that were not fully adjusted (**Figures 5**, **6**).

### 3.4 Association between genotype and blood glucose level

In overall, no significant association was found between any genotype and blood glucose levels in any SNP (**Supplementary Table S2**). The 1-hour PG level of the TC genotype of rs1111875, however, were considerably greater than those of the TT genotype in participants over 30 years of age (*P* < 0.05, **Table 3**). In the pre-BMI < 18.5 group, those with the TC genotype of rs1111875 had a substantially higher 2-hour postprandial glucose level than people with the CC genotype (*P* < 0.05). Furthermore, people with the CC genotype of rs5015480 had a substantially lower 2-hour postprandial glucose level than people with the TT and TC genotypes (*P* < 0.05, **Table 3**). Individuals with the CC genotype of rs5015480 had a lower FBG level in the pre-BMI ≥ 24 group than those with the TC and TT genotypes (*P* < 0.05, **Table 3**). Furthermore, PG levels at 1 and 2 hours were considerably greater in persons with the TA genotype of rs9939609 than in those with the TT genotype (*P* < 0.05, **Table 3**). No significant association was found between genotype and blood glucose levels in other groups (*P* > 0.05, **Supplementary Table S2**).

**Table 3:** Association between genotype and blood glucose level, gestational week of labor and neonatal weight.

Groups	SNP	Genotype	FBG (mmol/L)	1 h-PG (mmol/L)	2 h-PG (mmol/L)	Gestational week of labor	Neonatal weight (g)
age ≥ 30	rs1111875	TT	4.710 ± 0.421	9.261 ± 1.777**^a^**	8.159 ± 1.730	38.84 ± 1.991	3240.56 ± 362.961
		TC	4.711 ± 0.407	9.815 ± 1.718**^a^**	8.528 ± 1.700	38.97 ± 1.638	3180.47 ± 436.814
		CC	4.680 ± 0.389	9.562 ± 1.910	8.589 ± 1.907	38.37 ± 1.416	3130.29 ± 371.606
		F	0.086	5.430	2.850	1.622	2.080
		*P*	> 0.05	< 0.05	> 0.05	> 0.05	> 0.05
pre-BMI < 18.5	rs1111875	TT	4.524 ± 0.371	8.467 ± 1.770	7.425 ± 1.500	39.24 ± 1.361	3099.34 ± 345.266
		TC	4.553 ± 0.638	8.718 ± 1.978	7.952 ± 1.734**^a^**	38.76 ± 3.848	3106.77 ± 320.460
		CC	4.423 ± 0.281	7.589 ± 1.261	6.384 ± 1.424**^a^**	39.56 ± 1.810	2938.89 ± 446.443
		F	0.289	1.530	4.478	0.786	0.980
		*P*	> 0.05	> 0.05	< 0.05	> 0.05	> 0.05
	rs5015480	TT	4.524 ± 0.371	8.508 ± 1.877	7.504 ± 1.524**^a^**	39.23 ± 1.288	3103.83 ± 338.107
		TC	4.527 ± 0.673	8.684 ± 1.722	7.910 ± 1.831**^b^**	38.47 ± 4.842	3101.84 ± 341.688
		CC	4.653 ± 0.196	6.893 ± 0.913	5.820 ± 1.260**^ab^**	40.00 ± 2.708	2695.00 ± 284.429
		F	0.132	1.739	3.320	1.475	2.847
		*P*	> 0.05	> 0.05	< 0.05	> 0.05	> 0.05
pre-BMI ≥ 24	rs5015480	TT	4.938 ± 0.470**^a^**	9.691 ± 1.921	8.174 ± 1.668	38.86 ± 1.349	3329.95 ± 338.066
		TC	4.853 ± 0.547**^b^**	9.761 ± 2.139	8.358 ± 2.128	39.03 ± 1.577	3341.50 ± 463.048
		CC	4.204 ± 0.102**^ab^**	9.598 ± 2.205	8.814 ± 1.865	38.20 ± 0.837	3374.00 ± 510.176
		F	5.485	0.025	0.383	0.804	0.040
		*P*	< 0.05	> 0.05	> 0.05	> 0.05	> 0.05
	rs9939609	TT	4.843 ± 0.410	9.443 ± 1.823**^a^**	8.036 ± 1.556^a^	39.00 ± 1.337	3313.61 ± 368.909
		TA	4.996 ± 0.701	10.445 ± 2.332**^a^**	8.791 ± 2.397**^a^**	38.50 ± 1.606	3418.75 ± 421.960
		AA	5.103 ± 0.849	10.690 ± 0.961	9.747 ± 1.190	39.00 ± 1.000	3176.67 ± 282.194
		F	1.386	3.506	3.176	1.574	1.199
		*P*	> 0.05	< 0.05	< 0.05	> 0.05	> 0.05

^a,b^A *p*-value<0.05 indicates statistical significance.

### 3.5 Association between genotype and gestational week of labor

In all groups, there was no discernible relationship between any genotype and the gestational week of labor (*P* > 0.05, **Table 3**; **Supplementary Table S2**).

### 3.6 Association between genotype and neonatal weight

In all groups, there were no significant differences between genotypes and newborn weight (*P* > 0.05, **Table 3**; **Supplementary Table S2**).

### 3.7 Rs11705701 meta-analysis results

**Figure 7:**
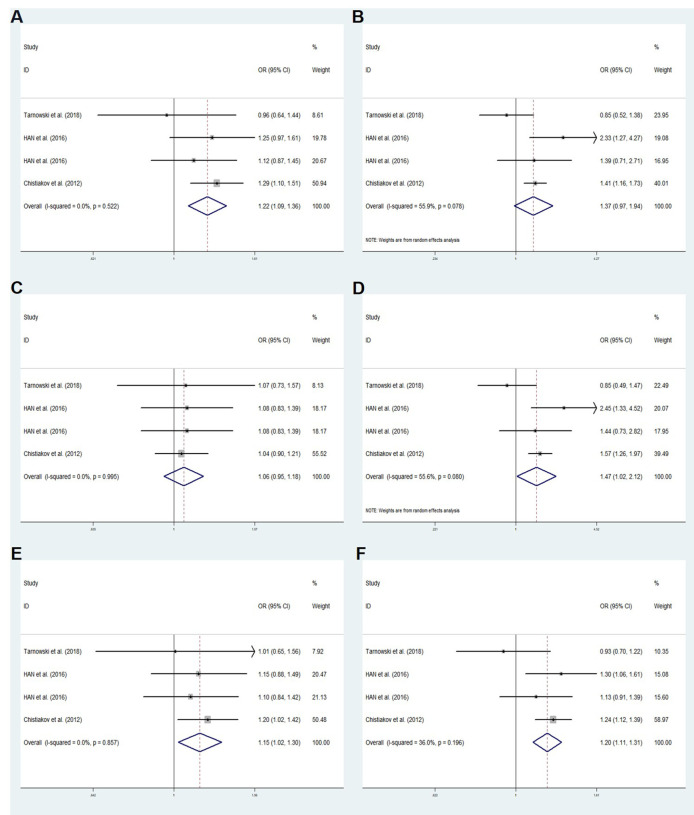
Meta-analysis for the association between the IGF2BP2 rs11705701 and GDM susceptibility. **(A)** Dominant model, AA+GA vs.GG (fixed effects mode); **(B)** Recessive model, AA vs.GA+GG (random effects model); **(C)** Overdominant model, GA vs.AA+GG (fixed effects model); **(D)** Homozygote model: AA vs. GG (random effects model); **(E)** Heterozygote model: GA vs. GG (fixed effects model); **(F)** Allele model, A vs. G (fixed effects model). OR, odds ratio; CI, confidence interval; I-squared, measure to quantify the degree of heterogeneity in meta-analyses.

**Figure 8:**
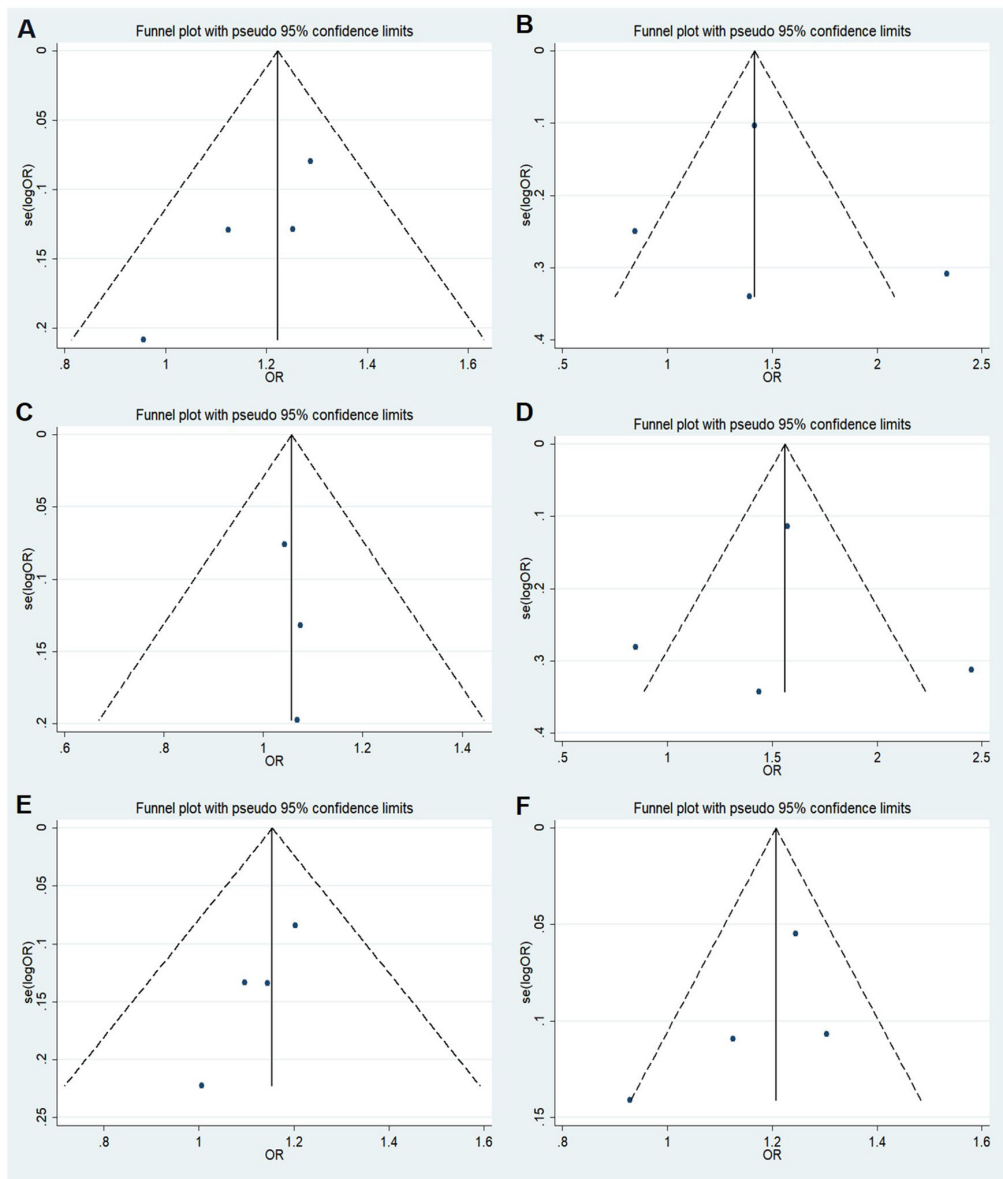
Funnel plot of the odds ratios in the meta-analysis. **(A)** Dominant model, AA+GA vs.GG; **(B)** Recessive model, AA vs.GA+GG; **(C)** Overdominant model, GA vs.AA+GG; **(D)** Homozygote model: AA vs. GG; **(E)** Heterozygote model: GA vs. GG; **(F)** Allele model, A vs. G.

We performed a meta-analysis of published research to gain a better understanding of the relationship between rs11705701 and diabetes because the control group in our study did not follow HWE. Four studies were included in the final analysis: two studies about rs11705701 and T2DM, one study about rs11705701 and GDM, and one study about rs11705701 and prediabetes mellitus (Pre-DM). The features of the research are displayed in **Supplementary Table S3**. **Figure 7** illustrates the associations found in the overall analysis between the various models and increased risk of diabetes mellitus. The dominant (AA+GA vs. GG: OR=1.218; 95% CI: 1.088-1.364; *P* = 0.001), homozygous (AA vs. GG: OR=1.472; 95% CI: 1.023-2.119; *P* = 0.037), heterozygous (GA vs. GG: OR = 1.153; 95% CI: 1.024-1.298; *P* = 0.019), and allele (A vs. G: OR=1.202; 95% CI: 1.106-1.307; *P* < 0.001) models demonstrated associations with increased risk of diabetes. In other groups, there was no discernible difference (*P* > 0.05, **Figure 7**). The funnel plot was shown to be symmetrical (*P* > 0.05, **Figure 8**). Egger’s tests yielded consistent results (all *P* > 0.05), indicating the absence of publication bias.

### 3.8 Transcriptional factor prediction outcomes

**Figure 9:**
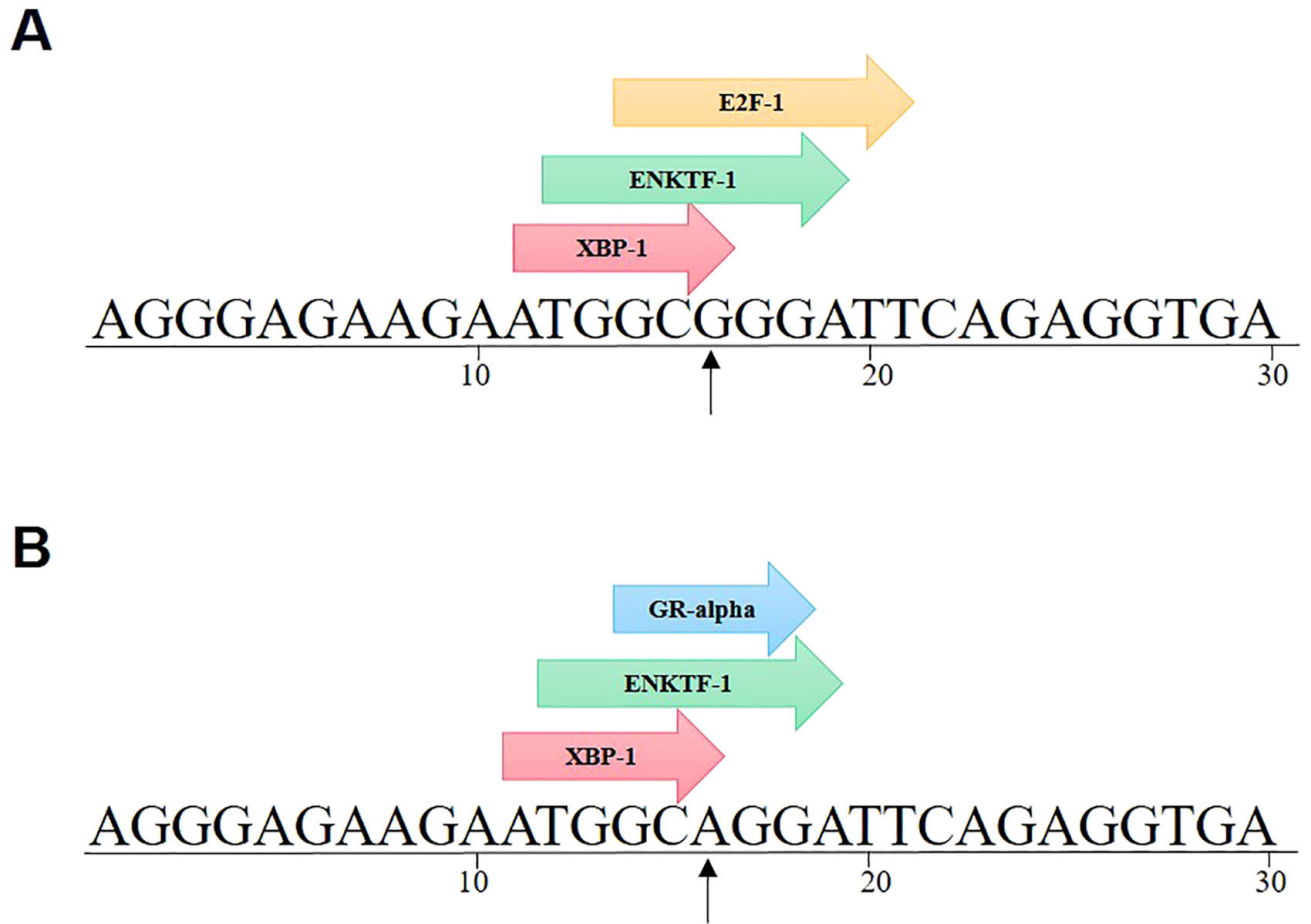
IGF2BP2 rs11705701 G > A transcription factor prediction. rs11705701 G > A affects the binding of GR-alpha and E2F-1 transcription factors. **(A)** rs11705701 (reference); **(B)** rs11705701 (mutant).

To investigate if SNPs in the IGF2BP2 gene’s promoter affect particular transcription factor binding locations, the PROMO database was consulted. SNPs have an impact on the binding of pertinent transcription factors, as seen in **Figure 9**. It was discovered that the binding of GR-alpha and E2F-1 transcription factors was impacted by rs11705701 G > A. It was discovered that the rs11705701 A allele binds exclusively to the GR-alpha transcription factor, while the rs11705701 G allele preferentially binds to the E2F-1 transcription factor.

## 4 Discussion

Variants in genetic composition can alter how encoded proteins are expressed and function, which can have a broad range of physiological effects. Thus, polymorphisms might be clinically significant for a range of diseases (25). A higher risk of GDM has been linked to alleles in HHEX, IGF2BP2, and FTO (22, 26). We studied the Chinese population’s susceptibility to GDM using HHEX rs1111875 and rs5015480, IGF2BP2 rs11705701 and rs4402960, and FTO rs9939609. **Figure 1** shows that there was a considerable increase in the likelihood of developing GDM due to the rs1111875 TC in HHEX. According to a recent meta-analysis, the rs1111875 CC and CT genotype group had a 50% and 29% higher risk of GDM than the TT genotype population, respectively (27). Similar to our results, Benny et al. discovered that HHEX rs1111875 was substantially related to GDM susceptibility (28). A meta-analysis revealed that the HHEX rs5015480 C allele was linked to GDM susceptibility, and the same rs5015480 polymorphism was found to be similarly associated with T2DM in a study of T2DM (29). Furthermore, we discovered that the overdominant model in HHEX rs5015480 was significantly associated with GDM in the age ≥ 30 years group.

The HHEX gene rs1111875 and rs5015480 have been identified as typical loci linked with diabetes since GWAS started to validate candidate gene research in various ethnic groups (12–15). The IDE, KIF11, and HHEX genes are found on human chromosome 10 and are positioned in the LD region at q23.33, which is home to the HHEX gene. The HHEX-KIF-IDE region, which is closest to the HHEX gene, has the C/T variants rs1111875 and rs5015480. These variants have been linked to pancreatic embryonic development and may have an impact on future insulin secretion. In this study, we observed a significant association between HHEX SNPs and susceptibility to GDM, particularly among individuals aged 30 years and older. Furthermore, individuals with the TC genotype exhibited significantly higher 1-hour glucose levels compared to the TT genotype. This finding suggests that genetic variations in the HHEX gene may contribute to β-cell dysfunction, thereby influencing the onset of diabetes mellitus. The HHEX rs1111875 is implicated in the Wingless-type MMTV integration site (WNT) signaling pathway, impacting susceptibility to diabetes. This SNP encodes transcription factors that modulate gene expression in cellular processes, influencing cell development and growth (30, 31). Similarly, among the Greek Cypriots, the HHEX rs5015480 has been linked to altered insulin secretion, cellular function, and diabetes susceptibility. The HHEX rs1111875 and rs5015480, have been associated with diabetes risk across diverse populations, primarily due to their role in reducing β-cell response and insulin secretion (30, 32–35). Importantly, HHEX gene polymorphisms are believed to influence insulin production and secretion (36, 37).

Dysregulation of IGF2BP2 has been linked to various metabolic diseases and cancers (38, 39). Notably, the IGF2BP2 SNP is associated with both T2DM and cancer (38). In 2007, Grarup et al. reported no association between IGF2BP2 gene variants and pancreatic cell dysfunction in a Danish cohort (40). Subsequent research revealed that IGF2BP2 variants diminish glucose-stimulated insulin secretion in the initial phase of diabetes progression, indicating an impact on pancreatic β-cell function (41–43). The IGF2BP2 rs11705701 G/A variant, situated in the -1479 locus of the promoter region, has been linked to reduced body fat and insulin resistance in Mexican Americans, elevating the susceptibility to T2DM (44). Moreover, this variant is associated with a heightened T2DM risk in the Russian population, with allele A correlating with a truncated IGF2BP2 protein in the adipose tissue of non-obese individuals (45). Furthermore, the rs11705701 variant exhibits a strong association with female prediabetic patients (46). While no significant association was observed between IGF2BP2 rs11705701 and the risk of GDM in the Polish population, it is linked to prolonged gestation and enhanced neonatal health, as indicated by Apgar scores (47). Collectively, these findings suggest a potential role for IGF2BP2 rs11705701 in diabetes development. Comparative analyses were not conducted with the control group in our study due to non-compliance with Hardy-Weinberg equilibrium. Nonetheless, through a meta-analysis of relevant previously published studies, we identified a significant association between the rs11705701 A variant and an increased risk of developing diabetes mellitus. Utilizing a transcription factor prediction analysis website, we further investigated the impact of rs11705701 variants on promoter activity. Our analysis revealed specific binding patterns, with rs11705701 G binding E2F1 and rs11705701 A binding GR-alpha transcription factor. This suggests that IGF2BP2 rs11705701 may regulate its transcriptional activity by modulating transcription factor binding. Subsequent molecular biology experiments are warranted to confirm these findings. Additionally, our results suggested that IGF2BP2 rs4402960 was not associated with the risk of GDM, consistent with recent studies and comprehensive quantitative meta-analyses (48–50).

FTO proteins function within the nucleus to remove N6-methyladenosine modifications from mRNA, thereby influencing the splicing of genes crucial for adipogenesis (51). Notably, variations located in the initial intron of the FTO gene have been linked to elevated BMI and T2DM, with a 47 kb genomic segment identified to harbor multiple SNPs associated with these conditions. Among these, the rs9939609 variant has been extensively researched (52). The precise mechanism through which this SNP contributes to obesity remains elusive. Nevertheless, individuals heterozygous for rs9939609 exhibit heightened levels of primary FTO transcripts in the risk A allele compared to the T allele (53), potentially resulting in increased FTO expression that promotes adipogenesis. Notably, this latter association has not been documented in existing literature. The FTO rs9939609, situated in the first intron of the gene, has been linked to a heightened risk of GDM in Caucasian populations. Our study revealed a significant association between carriers of the A allele and an increased risk of GDM in individuals aged ≥ 30. Furthermore, the A allele was found to be correlated with accelerated weight gain during pregnancy. Additionally, our investigation demonstrated elevated 1-hour and 2-hour glucose levels in the OGTT among individuals with the TA genotype compared to the TT genotype in the pre-BMI ≥ 24 group. But some studies describing the absence of association of FTO (rs9939609) and GDM risk (49, 50), this was consistent with our overall analysis results. These genetic variations may potentially impact FTO expression or enzyme activity, resulting in metabolic alterations that disrupt glucose metabolism and induce insulin resistance, consequently heightening the susceptibility to GDM.

The modest sample size of the GDM and control groups necessitates validation of our observations in a larger cohort in future studies. The scope of the study was limited to Chinese individuals, underscoring the need for further research to confirm our findings in diverse populations.

## 5 Conclusions

In conclusion, our study revealed an elevated risk of GDM associated with the TC genotype of the HHEX gene rs1111875, particularly among individuals aged ≥ 30. Additionally, rs5015480 and rs9939609 showed significant correlations with GDM in the same age group. These findings suggest a potentially stronger link between these specific SNPs and GDM among women of advanced maternal age.

## Data Availability

The datasets presented in this study can be found in online repositories. The names of the repository/repositories and accession number(s) can be found in the article/**Supplementary Material**.

## Supplementary material

Supplementary Figure 1 The associations between rs1111875 and GDM risk in different pre-BMI subgroups. *adjusted.

Supplementary Figure 2 The associations between rs5015480 and GDM risk in different pre-BMI subgroups. *adjusted.

Supplementary Figure 3 The associations between rs9939609 and GDM risk in different pre-BMI subgroups. *adjusted.
