# Supplementary figures and images for: Association between genetic polymorphisms and gestational diabetes mellitus susceptibility in a Chinese population
Source: Front Endocrinol (Lausanne). 2024 Nov 26;15:1397423. doi: 10.3389/fendo.2024.1397423 (PMC11628248; doi:10.3389/fendo.2024.1397423)

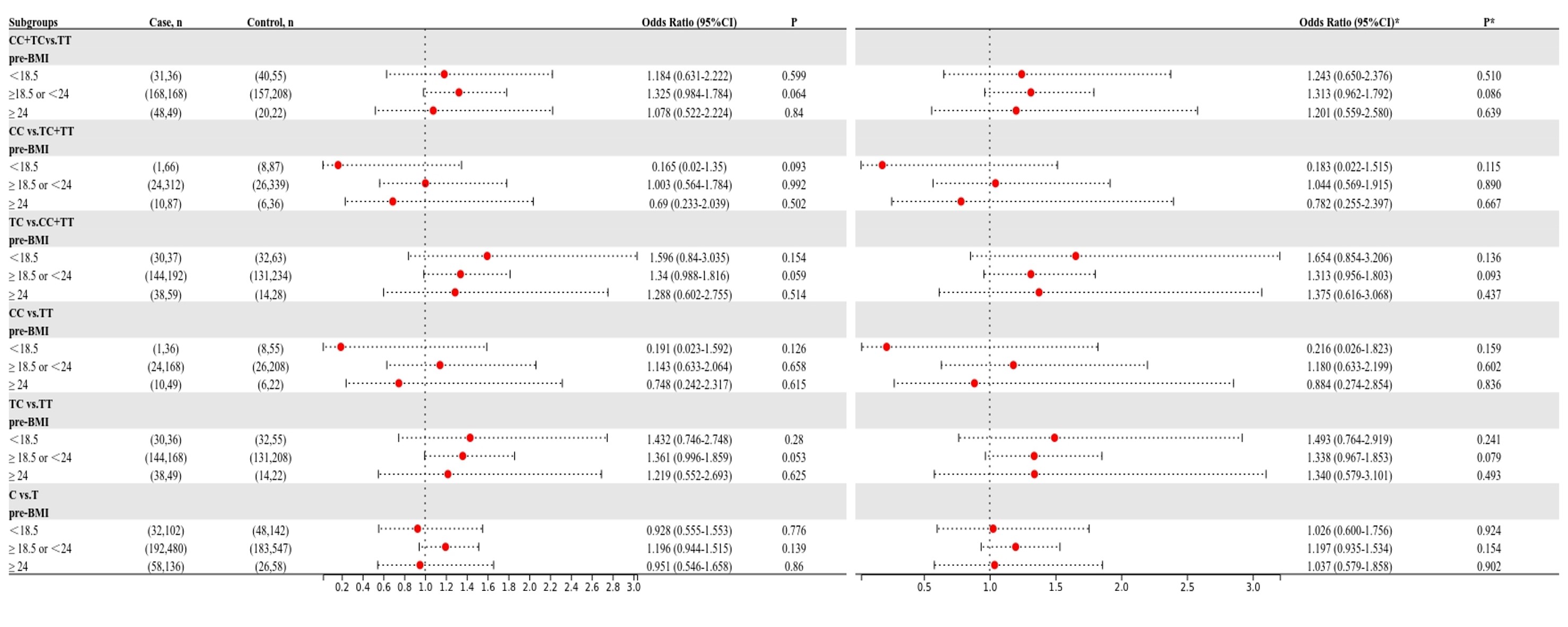

Supplement: Supplementary Figure 1 — The associations between rs1111875 and GDM risk in different pre-BMI subgroups. *adjusted. [file Image1.jpeg]

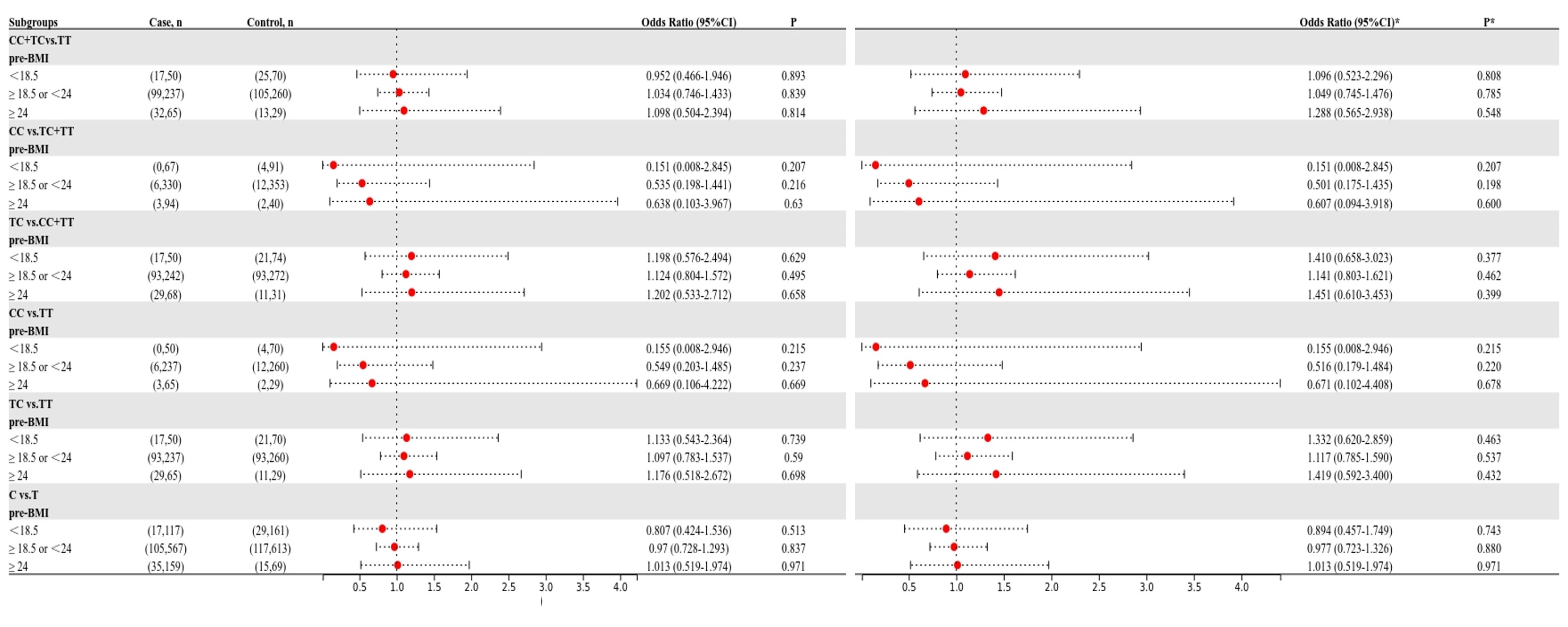

Supplement: Supplementary Figure 2 — The associations between rs5015480 and GDM risk in different pre-BMI subgroups. *adjusted. [file Image2.jpeg]

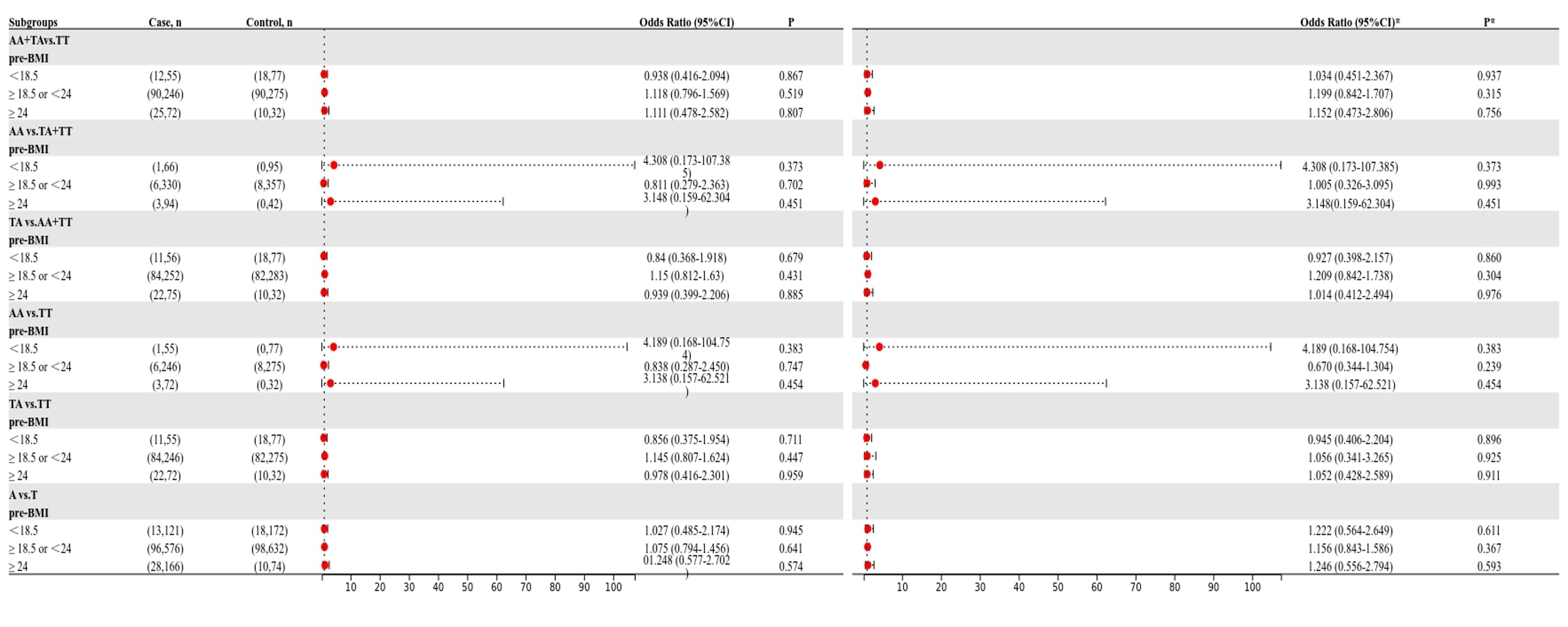

Supplement: Supplementary Figure 3 — The associations between rs9939609 and GDM risk in different pre-BMI subgroups. *adjusted. [file Image3.jpeg]
